# A CT-based nomogram for differentiating invasive fungal disease of the lung from bacterial pneumonia

**DOI:** 10.1186/s12880-022-00903-5

**Published:** 2022-10-03

**Authors:** Meilin Gong, Jingmei Xu, Kang Li, Ke Li, Yuwei Xia, Yang Jing, Jiafei Chen, Jing Li, Jing Yang, Mingshan Du, Wenjing Hou, Yuan Ou, Lian Li, Wei Chen

**Affiliations:** 1 Department of Radiology, The First Affiliated Hospital, Army Medical University, 30 Gaotanyan Street, Shapingba District, Chongqing, 400038 China; 2 Department of Radiology, Chongqing General Hospital, Chongqing, China; 3 Department of Radiology, The First Affiliated Hospital of Chongqing Medical University, Chongqing, China; 4 Department of Radiology, Sichuan Provincial Corps Hospital of Chinese People’s Armed Police Force, LeShan, China; 5 Huiying Medical Technology Co., Ltd. Room C103, B2, Dongsheng Science and Technology Park, Beijing, China

**Keywords:** Lung, Invasive fungal disease, Bacterial pneumonia, Radiomics, Nomogram

## Abstract

**Background:**

There is an annual increase in the incidence of invasive fungal disease (IFD) of the lung worldwide, but it is always a challenge for physicians to make an early diagnosis of IFD of the lung. Computed tomography (CT) may play a certain role in the diagnosis of IFD of the lung, however, there are no specific imaging signs for differentiating IFD of lung from bacterial pneumonia (BP).

**Methods:**

A total of 214 patients with IFD of the lung or clinically confirmed BP were retrospectively enrolled from two institutions (171 patients from one institution in the training set and 43 patients from another institution in the test set). The features of thoracic CT images of the 214 patients were analyzed on the picture archiving and communication system by two radiologists, and these CT images were imported into RadCloud to perform radiomics analysis. A clinical model from radiologic analysis, a radiomics model from radiomics analysis and a combined model from integrating radiologic and radiomics analysis were constructed in the training set, and a nomogram based on the combined model was further developed. The area under the ROC curve (AUC) of the receiver operating characteristic (ROC) curve was calculated to assess the diagnostic performance of the three models. Decision curve analysis (DCA) was conducted to evaluate the clinical utility of the three models by estimating the net benefit at a range of threshold probabilities.

**Results:**

The AUCs of the clinical model for differentiating IFD of lung from BP in the training set and test sets were 0.820 and 0.827. The AUCs of the radiomics model in the training set and test sets were 0.895 and 0.857. The AUCs of the combined model in the training set and test setswere 0.944 and 0.911. The combined model for differentiating IFD of lung from BP obtained the greatest net benefit among the three models by DCA.

**Conclusion:**

Our proposed nomogram, based on a combined model integrating radiologic and radiomics analysis, has a powerful predictive capability for differentiating IFD from BP. A good clinical outcome could be obtained using our nomogram.

**Supplementary Information:**

The online version contains supplementary material available at 10.1186/s12880-022-00903-5.

## Introduction

The increase in the incidence of hematological malignancies, clinically invasive operations, and use of corticosteroids and immunosuppressants has led to an annual increase in the incidence of invasive fungal disease (IFD) of the lung, a condition with a high mortality in patients with hematological malignancies and organ transplantation [1] worldwide. Early detection of the disease and timely clinical drug intervention can significantly improve patient prognosis and reduce mortality. However, early diagnosis is challenging because the clinical symptoms of IFD of the lung show no obvious specificity. Puncture biopsy is an invasive examination and thus difficult for some patients to accept. The serum 1,3-β-D-glucan test and galactomannan (GM) test are helpful in obtaining a diagnosis but have low sensitivity and an undesirable false-positive rate. High-resolution chest computed tomography (CT) plays an important role in the diagnosis of lung diseases. The European Organization for Research and Treatment of Cancer and the Mycoses Study Group Education and Research Consortium (EORTC/MSGERC) consensus definitions of IFDs include the key role of CT scans [2], indicating that it is the most important tool for early management of IFD. However, the CT features of IFD are nonspecific, as other diseases of the lung have similar CT manifestations, especially the most common infectious disease, bacterial pneumonia (BP). Thus, radiologists have difficulty accurately diagnosing IFD in practice.

In recent years, newly emerging radiomics methods have emphasized that radiological images contain not only visual data that can be observed by the naked eye but also implicitly abundant genetic information [3]. High-throughput extraction and analysis of quantitative features from medical images by sophisticated machine learning tools are used to transform medical images into minable data; these techniques have been mainly applied in tumor research. However, research on nontumor lesions using radiomics methods is relatively limited. Wang Yanling [4] studied the CT differential diagnosis of pneumonia and pulmonary paraquat poisoning with radiomics methods, demonstrating that the resulting predictive model had high differential diagnostic performance. In the present study, we aimed to distinguish IFD from BP by combining radiologic and radiomics analysis to achieve the early diagnosis of IFD.

## Methods

### Patients

The Ethics Committee of the First Affiliated Hospital of Army Medical University approved this retrospective study and waived informed consent from the patients due to the retrospective nature of the study. All methods in the study were performed in accordance with the 2002 Declaration of Helsinki.

**Fig. 1:**
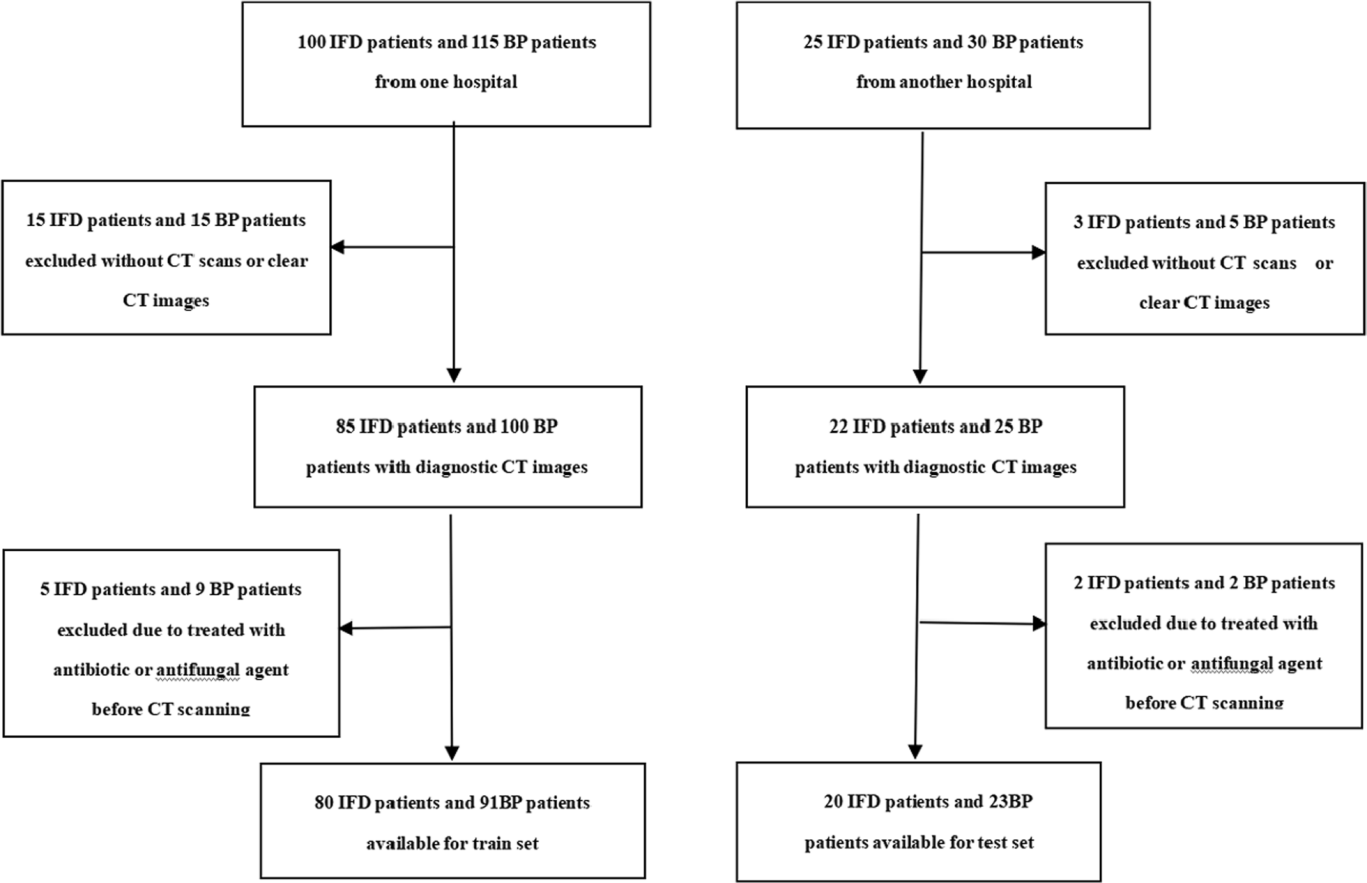
Flow chart of enrolling patients in the study

A total of 80 patients with IFD of the lung and 91 patients with clinically confirmed BP admitted to the First Affiliated Hospital of Army Medical University from October 2014 to September 2015 were enrolled retrospectively and considered the training set. An additional 20 patients with IFD of the lung and 23 patients with BP confirmed clinically admitted to the First Affiliated Hospital of ChongQing Medical University from January 2018 to June 2018 were enrolled retrospectively and considered the test set in this study. The pathogen categories and numbers of patients with each pathogen are shown in Table 1. The inclusion criteria were as follows: (1) patients with proven or probable IFD according to the EORTC/MSGERC criteria [2, 5]: Proven IFD was defined by histopathological evidence of tissue invasion. Probable IFD was defined based on the presence of host factors, clinical findings such as a halo sign, air-crescent sign, cavity or consolidation on CT, and mycological evidence of fungal infection from culture, cytological analysis of bronchoalveolar lavage (BAL) fluid, or galactomannan measurement in the serum or BAL. (2) patients with a diagnosis of BP based on symptoms of respiratory infection, and consolidation on thoracic CT, in addition to positive sputum, bronchoscopic aspirate, blood or pleural fluid cultures. The exclusion criteria were as follows: (1) patients underwent treatment with antibiotics or antifungal agents before CT scanning; and (2) unclear lesion visualization on CT images. The flow chart of enrolling patient in the study is shown in Fig. 1.

**Table 1:** The pathogen categories and numbers of patients with each pathogen in the training set and test set

	IFD				BP		
Training set (n = 171)	Aspergillus	Candida	Others	Total	Gram-positive bacterium	Gram-negative bacterium	Total
	62	10	8	80	49	42	91
Test set (n = 43)	13	4	3	20	13	10	23

### CT image acquisition

The entire thorax of the patient was examined at the end of inspiration during a single breath-hold with a dual-source CT scanner (Somatom Definition, Siemens, Germany) at both institutions. The scanning parameters are shown in Table 2. Unenhanced CT images were acquired.

**Table 2:** The CT protocol of the two institutions

	Tube voltage (kVp)	Tube current (mAs)	Beam pitch	Detector collimation (mm)	Routine (matrix)	Slice thick (mm)	Slice interval (mm)
Training set	120	100–120	1	1.25	512 × 512	1.25	1
Test set	140	80–120	1	0.6	512 × 512	1.0	0.8

### Construction of models

#### Construction of the clinical model

##### Radiologic analysis of CT images

The CT images were interpreted using the picture archiving and communication system (PACS) of each institution. Blinded to the clinical information, two radiologists (reader 1 with 8 years of thoracic imaging experience and reader 2 with 10 years of thoracic imaging experience) analyzed the CT images and made decisions about the CT features by consensus, including lesion patterns, halos or the reverse halo sign (RHS), cavities, pleural effusion and lymph node enlargement. The lesion pattern was classified on CT as consolidation, nodules, ground glass opacity (GGO) or combinations thereof [6]. A nodule was considered a round or round-like lesion regardless of diameter (including tree-in-bud). Lymph node enlargement was defined as a short diameter of more than 1 cm along the short axis.

##### Construction of the clinical model

Univariable analysis was conducted to assess the differences in the above CT features between IFD of the lung and BP in the training set, and stepwise multivariable logistic regression analysis was performed on those CT features with statistically significant differences in the univariable analysis. Features with statistically significant differences in the multivariable analysis were considered independent risk factors related to the differential diagnosis of IFD of the lung and BP and were applied to build a model (named the clinical model). The odds ratio (OR) was obtained for each risk factor, and the area under the receiver operating characteristic (ROC) curve (AUC) was used to assess the diagnostic performance of the model with both the training and test sets.

#### Construction of the radiomics model

**Fig. 2:**
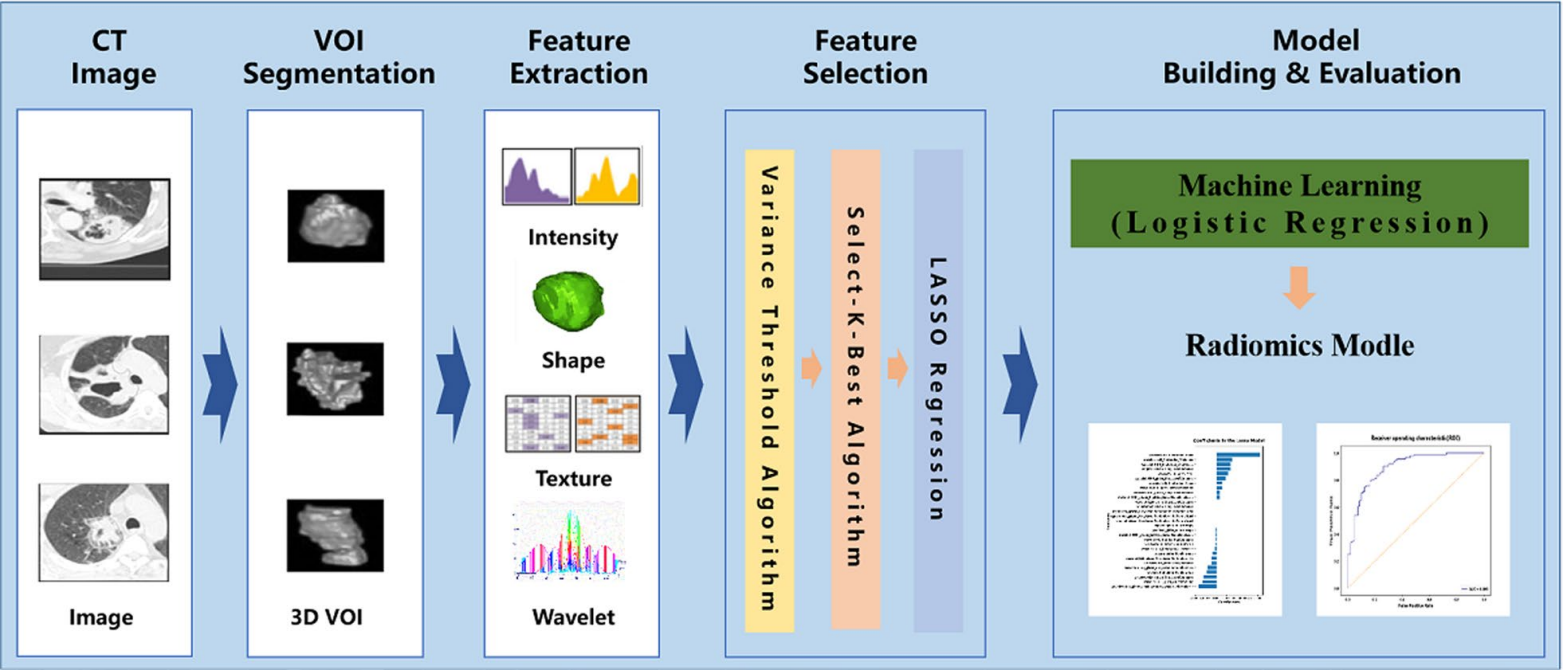
The workflow of the radiomics analysis of pneumonia

The workflow of the radiomics analysis of pneumonia is shown in Fig. 2.

##### CT image resample and segmentation

To minimize the discrepancies in the different image acquisition parameters and improve the reproducibility, we resampled all voxels into 1.0 mm × 1.0 mm × 1.0 mm with B-Spline interpolation from the raw data before image analysis, and normalized voxel values by subtracting its mean value and dividing by its standard deviation value.

CT images of all the patients from the PACS were imported into Dr. Turing AI-assisted diagnosis platform V3.0 (Huiying Medical Technology Co., Ltd., Beijing, China) to automatically segment pulmonary lesions presumed to be pneumonia (e.g., GGO, nodules, tree-in-bud and consolidation), and these segmentations were reviewed and corrected mannually (delineating around the edge of the lesions, avoiding vessels, bronchia, fibrosis and calcification) slice by slice by the same two radiologists to obtaine the volumes of interest (VOIs) based on entire lesions. AI-assisted automatic segmentation is an open source pneumonia deep learning segmentation model trained by Huiying on the platform. This model was trained from thousands of pneumonia cases by professional doctors who annotated the lesions, and the model adopts the Unet framework.

##### Radiomics feature extraction and selection

The segmented CT images were imported into the RadCloud platform (Huiying Medical Technology Co., Ltd., Beijing, China) to extract radiomics features. High-throughput data features were extracted from VOIs on the platform. These features were grouped into four groups. Group 1 (first-order statistics features) consisted of descriptors that quantitatively delineated the distribution of voxel intensities within the CT image through commonly used and basic metrics. Group 2 (shape- and size-based features) consisted of three-dimensional features that reflected the shape and size of the region. Group 3 (texture features) contained gray-level co-occurrence matrix (GLCM), gray-level size zone matrix (GLSZM), gray-level dependence matrix (GLDM), neighborhood gray-level dependence matrix (NGLDM) and gray-level run length matrix (GLRLM) that quantified regional heterogeneity differences. Group 4 (higher-order statistical features) included the first-order statistics and texture features derived from wavelet transformation of the original image. All radiomics features were standardized using the StandardScaler function by removing the mean and dividing by its standard deviation, and each set of feature values was converted to a mean of 0 with a variance of 1.

The inter-class and intra-class correlation coefficients (ICCs) were calculated to evaluate the reliability and reproducibility of features extracted from VOIs. CT images of 40 patients (20 IFDs and 20BPs) were chosen randomly, which were segmented automatically by AI-assisted diagnosis platform. Reader 1 and Reader 2 reviewed and corrected mannually VOIs independently, then Reader 1 repeated it a week later to evaluate the agreement of extracted features. Features with good agreement (ICCs > 0.75) were selected for further analyses. The VOIs reviewing and correcting of remaining image were conducted by Reader 1.

To reduce the redundant features, the variance threshold algorithm (variance threshold = 0.8) and Select-K-Best algorithm were adopted. The Select-K-Best algorithm used P < 0.05 to determine optimal features. To guarantee image feature robustness, the optimal features were selected by the least absolute shrinkage and selection operator (LASSO) method to best predict IFD of the lung and BP. In the LASSO method, the optimal alpha which is the coefficient of regularization was selected using inner tenfold cross-validation in the training set with the maximum iteration of 5000 via minimum average mean square error (MSE). Subsequently, the radiomics features with non-zero coefficients in the LASSO model generated by the whole training set with the optimal alpha were selected, which were used to build the radiomics model and calculate the radiomics score (Rad-score).

##### Construction of the radiomics model

A widely used support vector machine (SVM) machine learning algorithm was applied to construct the radiomics model by combining the selected radiomics features for the training set. The Rad-score was calculated as a linear combination of selected features weighted by LASSO coefficients. The AUC was used to assess the diagnostic performance of the radiomics model.

#### Construction of the combined model

A combined model was constructed by integrating radiologic and radiomics analysis of the training set, and a nomogram was further developed. The probability of detecting IFD of the lung for each patient was calculated using a nomogram-defined score for the training and test sets. The diagnostic performance of the nomogram in differentiating IFD of the lung from BP was assessed by the AUC with both the training and test sets. The calibration curve, along with a Hosmer–Lemeshow test, was constructed to assess the agreement of the nomogram-predicted probability and the real outcomes for IFD of the lung in both the training and test sets. The diagnostic performance of the three models was compared. Decision curve analysis (DCA) was conducted to estimate the clinical utility of the three models by calculating the net benefit at a range of threshold probabilities for both the training and test sets.

### Statistical analysis

We used RadCloud (Huiying Medical Technology Co., Ltd., Beijing, China) to perform radiomics statistical analysis. The radiologic analyses for images were performed with SPSS 23.0 and MedCalc15.2.2. The nomogram analysis was conducted with R software (version 3.3.1). The t test or Mann–Whitney U test was used for analysis of quantitative data, and the chi-square test or Fisher’s exact test was performed for analysis of qualitative data. The Delong test was applied to compare the diagnostic performance of the three models. A P value < 0.05 was considered statistically significant.

## Results

### Patient characteristics

There were no significant differences in age, sex or clinical diagnosis between the training and test sets (Table 3).

**Table 3:** Comparison of the training set and test set in terms of age, sex and clinical diagnosis

Clinical information		Training set	Test set	t/χ2 value	P value
Age (years)		50.3 + 19.4	49.6 + 21.5	0.227	0.821
Sex	Male	89	22	0.011	0.917
	Female	82	21		
Clinical diagnosis	BP	91	23	0.001	0.975
	IFD	80	20		

#### Construction of the clinical model

**Fig. 3:**
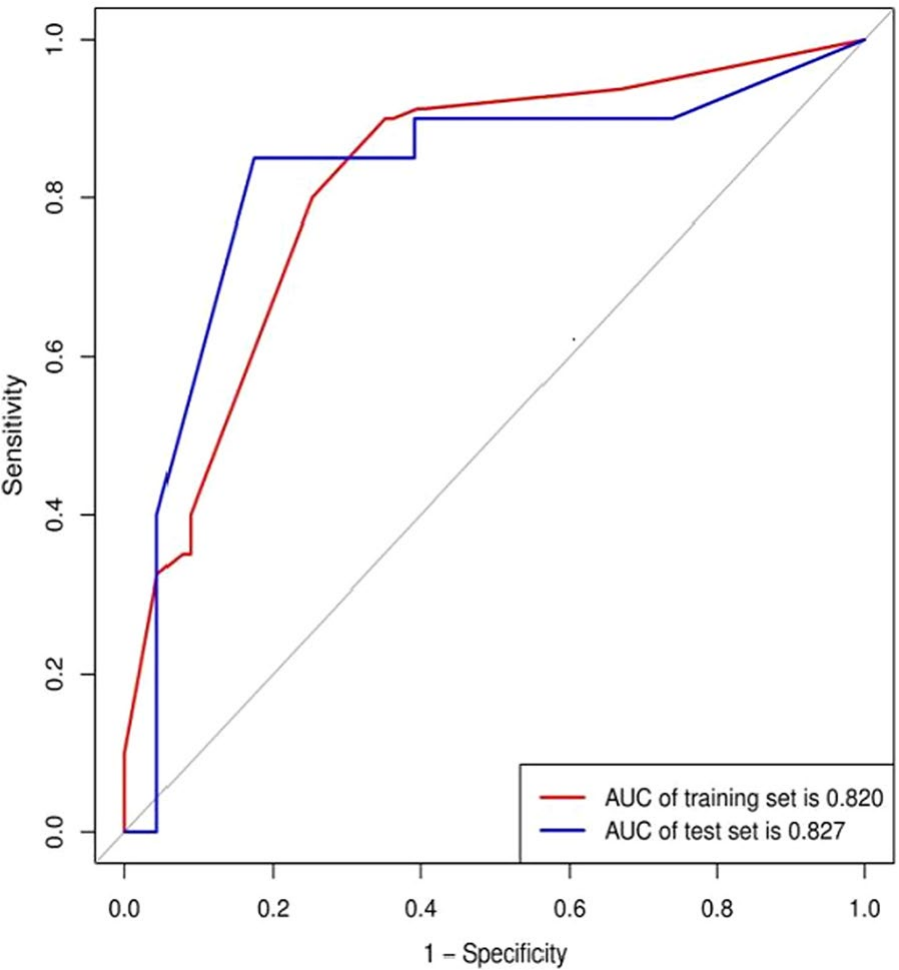
ROC curves of the clinical model for differentiating IFD of the lung from BP in the training and test sets

The comparison of CT features analyzed by the two radiologists between the IFD of the lung and BP groups in the training and test sets is shown in Table 4. There was a significant difference in the presence of patterns, halos or RHS and pleural effusion (P < 0.05) between the two groups, while there was no significant difference in the presence of lymph node enlargement and cavities, patient age or sex (P > 0.05) between the two groups in the training set. Multiple logistic regression analysis showed that only the pattern [odds ratio (OR) 3.157; 95% confidence interval (CI) 2.094–4.759; P < 0.001] and halo or RHS (OR 0.256; 95% CI 0.087–0.752; P = 0.013) remained independent predictors in the clinical model. The clinical model yielded AUC values of 0.820 in the training set and 0.827 in the test set (Fig. 3 and Table 6).

**Table 4:** Comparison of clinical factors in the training set

CT factor	Classification	IFD	BP	t/χ2 value	P value
Age (year)		53.1 + 18.2	47.9 + 20.2	1.776	0.077
Gender	Male Female	42 38	47 44	0.012	0.911
Pattern	Consolidation Nodule Combination GGO	7 3 67 3	56 3 29 3	57.248	< 0.001
Halo or RHS	Present Absent	25 55	5 86	19.5	0.001
Cavitation	Present Absent	24 56	27 64	0.002	0.962
Pleural effusion	Present Absent	16 64	41 50	12.0	0.001
LNE*	Present Absent	16 64	18 73	0.001	0.971

LNE* lymph node enlargement

#### Construction of the radiomics model

**Fig. 4:**
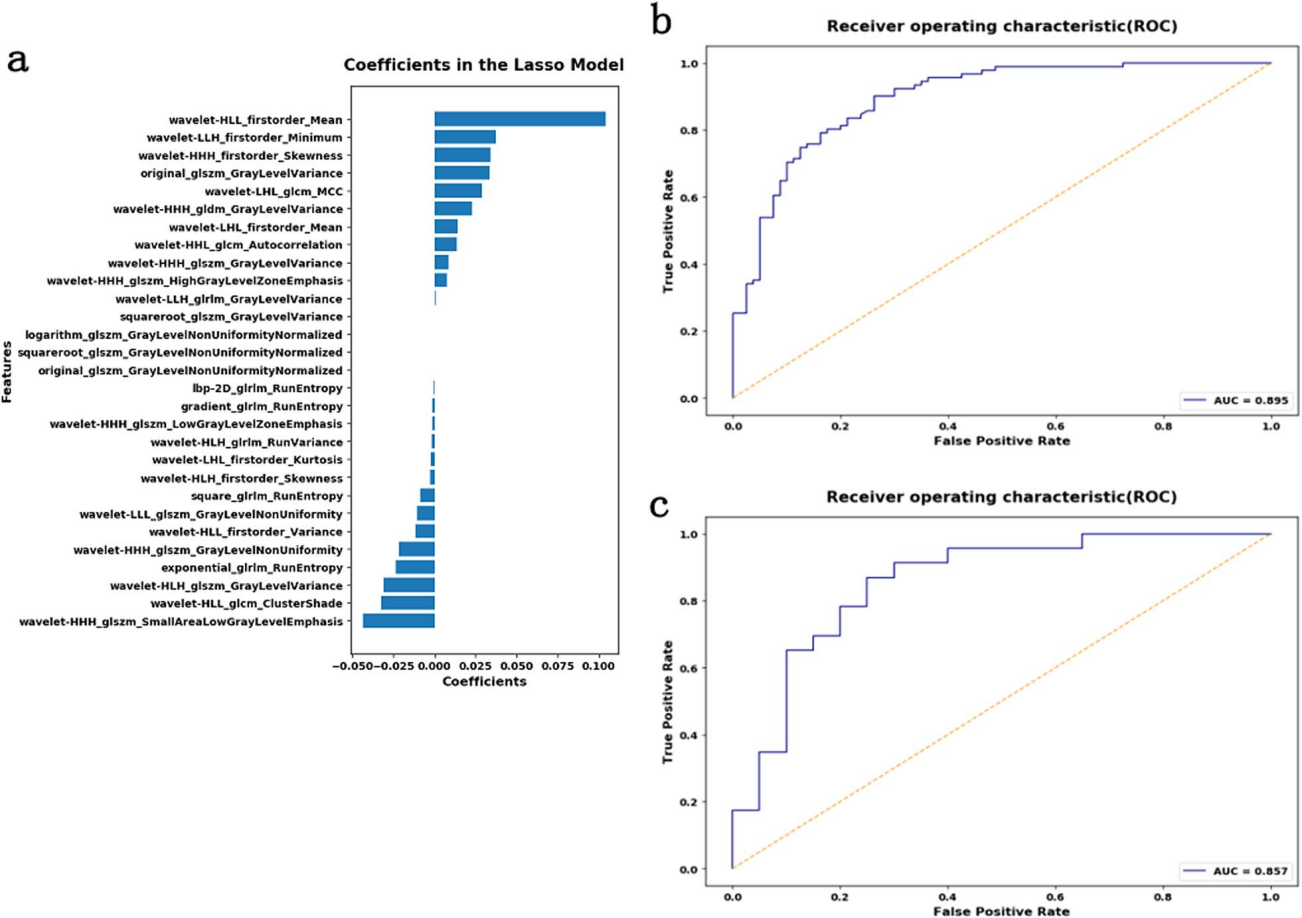
Coefficients of features in the radiomics model and ROC curves of the radiomics model for differentiating IFD of the lung from BP. **a** Coefficients in the LASSO model in the training set. **b** ROC curve of the radiomics model in the training set. **c** ROC curve of the radiomics model in the test set

A total of 1409 radiomics features were extracted from the Radcloud platform, containing 18 first-order statistics features, 14 shape- and size-based features, 75 texture features and 1302 higher-order statistics features, 29 optimal features of which were selected from the training set to construct the radiomics model, the coefficients of which are shown in Fig. 4 and Table 5.

**Table 5:** Features and coefficients of features in radiomics mode

Feature	Coefficient
Wavelet-HHH_glszm_SmallAreaLowGrayLevelEmphasis	− 0.0436137
Wavelet-HLL_glcm_ClusterShade	− 0.03231882
Wavelet-HLH_glszm_GrayLevelVariance	− 0.030891294
Exponential_glrlm_RunEntropy	− 0.023351974
Wavelet-HHH_glszm_GrayLevelNonUniformity	− 0.02186289
Wavelet-HLL_firstorder_Variance	− 0.011402046
Wavelet-LLL_glszm_GrayLevelNonUniformity	− 0.010425661
Square_glrlm_RunEntropy	− 0.008676379
Wavelet-HLH_firstorder_Skewness	− 0.002468136
Wavelet-LHL_firstorder_Kurtosis	− 0.00214606
Wavelet-HLH_glrlm_RunVariance	− 0.001889215
Wavelet-HHH_glszm_LowGrayLevelZoneEmphasis	− 0.001064299
Gradient_glrlm_RunEntropy	− 0.000995591
Ibp-2D_glrlm_RunEntropy	− 0.000411688
Original_glszm_GrayLevelNonUniformityNormalized	− 0.00000000599
Squareroot_glszm_GrayLevelNonUniformityNormalized	− 0.0000000013
Logarithm_glszm_GrayLevelNonUniformityNormalized	− 0.0000001918
Squareroot_glszm_GrayLevelVariance	0.00000195
Wavelet-LLH_glrlm_GrayLevelVariance	0.000633175
Wavelet-HHH_glszm_HighGrayLevelZoneEmphasis	0.007441552
Wavelet-HHH_glszm_GrayLevelVariance	0.008337091
Wavelet-HHL_glcm_Autocorrelation	0.013836544
Wavelet-LHL_firstorder_Mean	0.014166634
Wavelet-HHH_gldm_GrayLevelVariance	0.023156664
Wavelet-LHL_glcm_MCC	0.029071883
Original_glszm_GrayLevelVariance	0.033603816
Wavelet-HHH_firstorder_Skewness	0.033906287
Wavelet-LLH_firstorder_Minimum	0.037375992
Wavelet-HLL_firstorder_Mean	0.10444296
Intercept(non-feature)	0.532163743

The radiomics model achieved satisfactory predictive performance, with AUC values of 0.895 in the training set and 0.857 in the test set (Fig. 3 and Table 6).

**Table 6:** The diagnostic performance of the clinical model, radiomics model and combined model

Model	Training set					Test set				
	AUC(95%CI)	Cutoff	Accuracy	Sensitivity	Specificity	AUC (95%CI)	Cutoff	Accuracy	Sensitivity	Specificity
CD*	0.820 (0.754–0.874)	0.512	0.766	0.9	0.648	0.827 (0.681–0.925)	0.598	0.837	0.850	0.826
RD*	0.895 (0.839–0.936)	0.465	0.825	0.901	0.738	0.857 (0.716–0.945)	0.5	0.814	0.87	0.75
CBD*	0.944 (0.898–0.973)	0.481	0.883	0.9	0.868	0.911 (0.784–0.976)	0.205	0.837	0.95	0.739

CD* clinical model, RD* radiomics model, CBD* combined model

The Rad-score showed significant differences between the IFD (0.399 ± 0.195) and BP groups (0.649 ± 0.114) in the training set (*P* < 0.001, *Z* = 10.074) and in the test set (IFD, 0.404 ± 0.238; BP 0.663 ± 0.077; P < 0.001, *Z* = 4.66).

#### Construction of the combined model

**Fig. 5:**
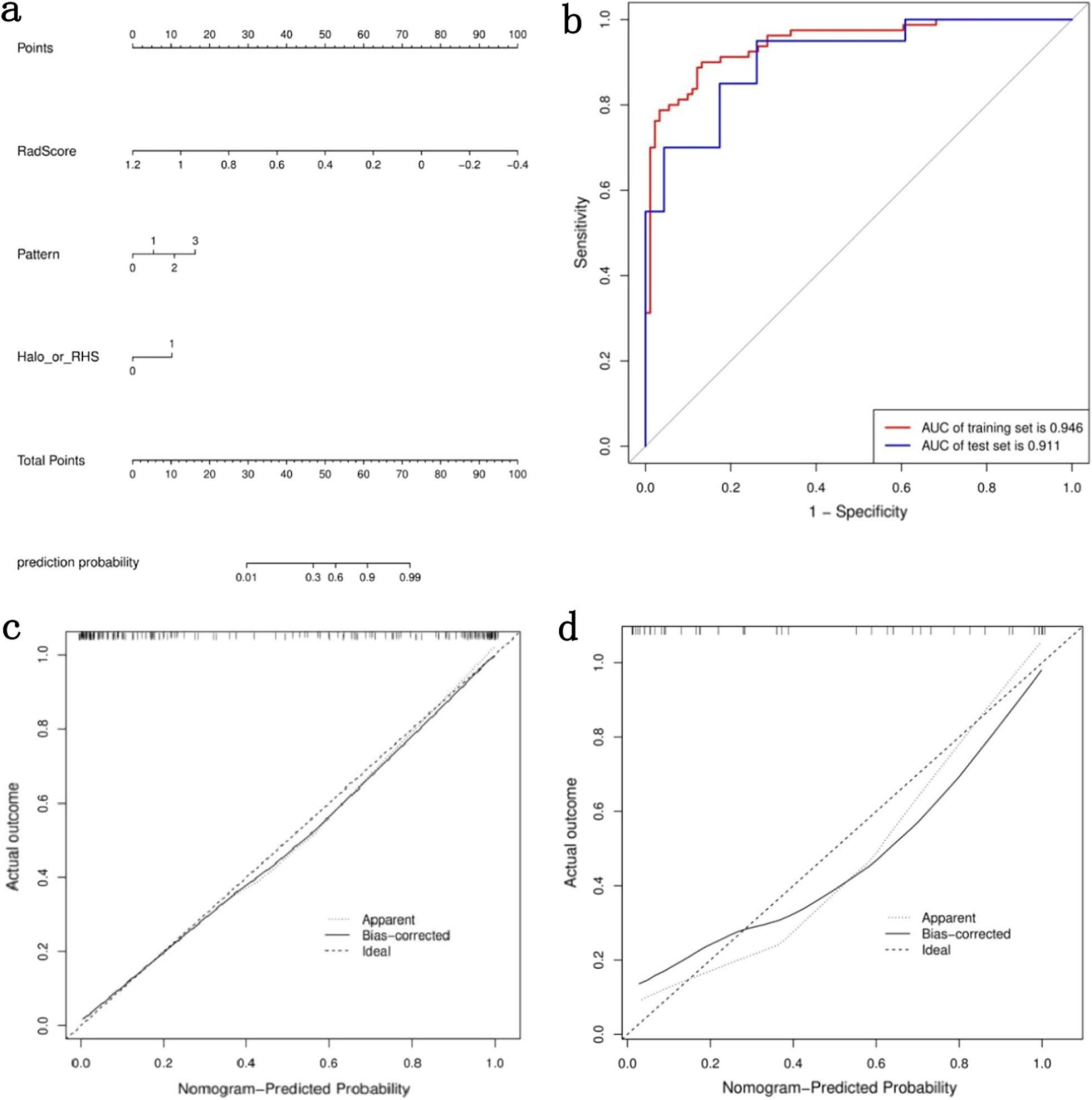
The nomogram of the combined model and its ROC curves and calibration curves. **a** The nomogram based on the combined model by integrating artificial analysis and the radiomic analysis in the training set. Pattern 0, 1, 3 and 2 represent consolidation, noddle, GGO and combinations thereof. Halo or RHS 0 and 1 represent absence and presence of Halo or RHS. Prediction probability is the estimated probability of IFD of the lung. **b** ROC curves of the combined model for differentiating IFD of the lung from BP in the training and test sets. **c** Calibration curves of the combined model for the training set and **d** calibration curves of the combined model for the test set. The y-axis represents the actual rate of IFD of the lung in the patients; the x-axis represents the nomogram-predicted probability of IFD of the lung. The Hosmer–Lemeshow test shows a good agreement of the nomogram with the perfect model represented by the black diagonal dashed line in both the training and test sets

**Fig. 6:**
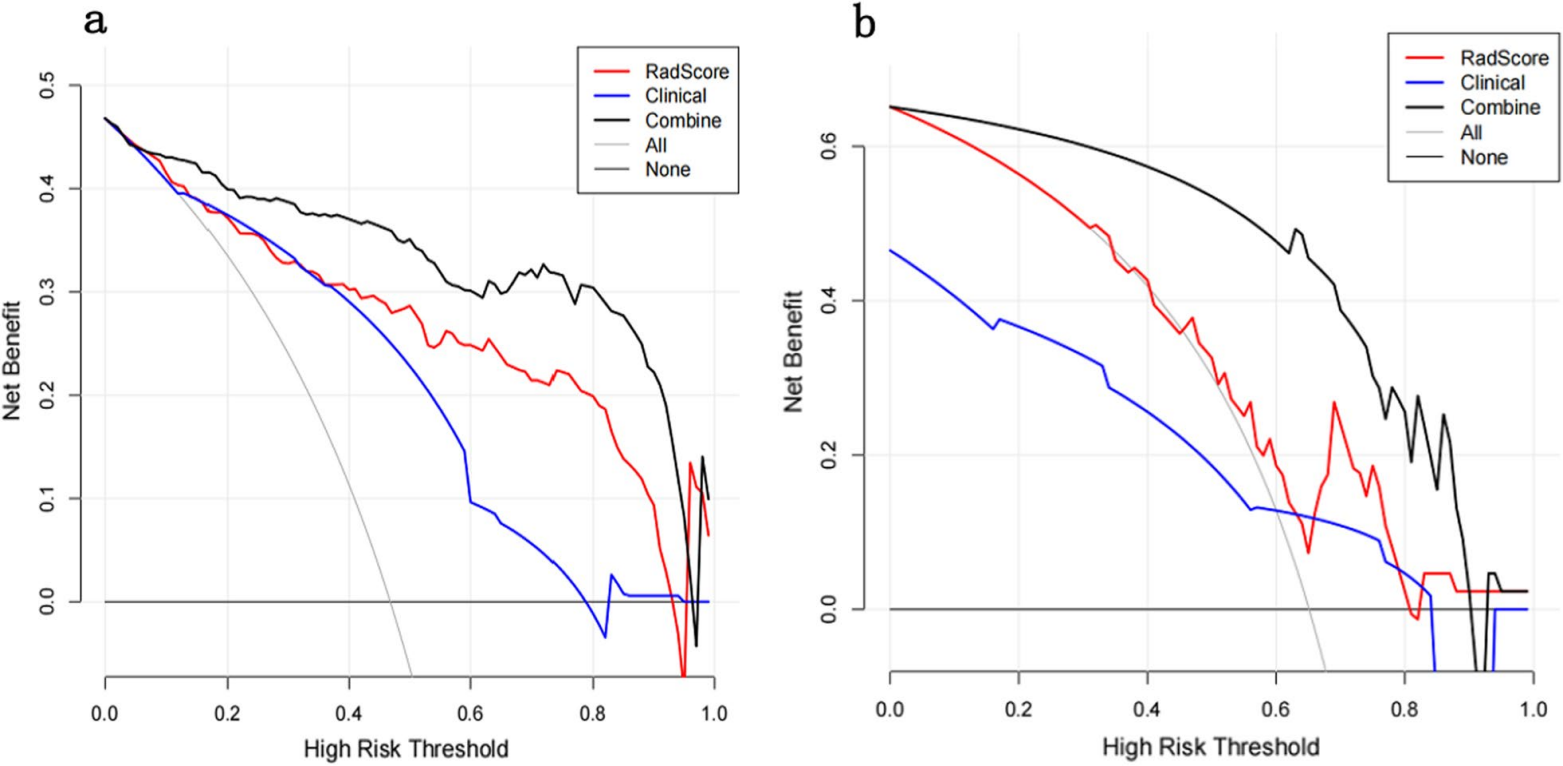
Decision curves for the combined model, clinical model and radiomics model. **a** Decision curves for three models in the training set, **b** decision curves for three models in the test set. The y-axis indicates the net benefit and the x-axis indicates threshold probability. The horizontal black line represents the assumption of all IFD patients, while the gray line represents the assumption of all BP patients. Based on the threshold probabilities obtained, the nomogram based on the combined model (black curve) provides the greatest net benefit among the three models in both the training and test sets

A nomogram was developed based on the combined model by integrating radiologic and radiomics analysis of the training set. The nomogram achieved optimal predictive performance, with AUC values of 0.944 in the training set and 0.911 in the test set. The calibration curve showed that there was great agreement between the nomogram-predicted probability and the real outcomes for IFD of the lung in both the training and test sets (Fig. 5 and Table 6). The Delong test showed that the combined model and the radiomics model had a significantly higher diagnostic performance than the clinical model (p = 0.025 and P < 0.001), whereas there was no significant difference between the combined model and the radiomics model (P = 0.087) in the training set, and no significant difference was found among the three models (radiomics model vs. clinical model, P = 0.792; combined model vs. clinical model, P = 0.107; radiomics model vs. combined model p = 0.280) in the test set. DCA showed that the nomogram based on the combined model provided higher net benefits than the clinical model and the radiomics model, which had the greatest clinical utility in differentiating IFD of the lung from BP among the three models (Fig. 6).

## Discussion

To improve the survival of patients with IFD of the lung and reduce their financial burden, early identification of IFD is essential, but this remains challenging for physicians. As revealed by our study, CT can help achieve this goal. The present study built a predictive diagnostic nomogram based on lung C T by integrating radiologic analysis with radiomics analysis for differentiating IFD of the lung from BP.

In the present study, the following five CT features of IFD of the lung and BP were analyzed by two radiologists: lesion pattern; halo sign or RHS; cavitation; pleural effusion; and lymph node enlargement. We found that the presence of a lesion pattern, halo sign or RHS and pleural effusion were significantly different between the two disease groups; thus, these CT features were subjected to multiple logistic regression analysis to establish a clinical model. Moreover, radiomics analysis was also used to build the radiomics signature. A combined model was further constructed by integrating the results of radiologic analysis and radiomic analysis of the training set, and a nomogram for differentiating IFD of the lung from BP was developed.

Among the CT features, significant differences were found between IFD and BP in terms of the lesion pattern, halo sign or RHS and pleural effusion, which is not completely consistent with a previous study [7] that showed that the halo sign was highly specific for invasive aspergillosis and that the presence of consolidation was not significantly different between IFD of the lung and BP. The halo sign is considered characteristic of angioinvasive aspergillosis [8–10], reflecting the presence of hemorrhagic microinfarcts, and is more common in the early phase. The RHS is also associated with IFD [11]. In the present study, 66 of the 137 patients with BP had pleural effusion, which is not consistent with a previous study reporting a frequency of 10% [12]. Because of these contradictory results, differentiating IFD of the lung from BP remains challenging for radiologists. Moreover, our established clinical model yields a sensitivity of 0.9 but a specificity of 0.65 in the training set, which is unreliable for wide use in clinical practice.

Radiomics analysis provides a quantitative measurement of heterogeneity through high-throughput extraction of image features, and it is not affected by subjective analysis. Radiomics analysis has been widely applied and demonstrated to be useful for studying tumor diagnosis and staging and evaluating prognosis [13–15]. According to these previous studies, tumor heterogeneity produces different texture features in radiologic images. In the present study, the radiomics method was used to establish a radiomics model for differentiating IFD of the lung from BP, which performed well in both the training set (AUC, 0.895) and the test set (AUC, 0.857). The potential reason for this good performance may be related to the heterogeneity of the two types of diseases; because they are caused by distinctly different pathogens, the image textures of these two diseases are different. Previous studies using CT radiomics [16, 17] predictive models have demonstrated great accuracy in the predictive diagnosis of COVID-19 pneumonia.

To explore the clinical usefulness of our findings, we established a nomogram based on the combined model as an individualized tool for predicting the risk of IFD for each pneumonia patient. Although there were differences in CT image acquisition protocol between the training and validation sets of the two institutions, the nomogram had an optimal capacity for prediction and generalization of the model, with a great diagnostic performance (AUC values of 0.944 in the training set and 0.911 in the test set). In addition to its great diagnostic performance, the nomogram demonstrated great net benefits for the majority of the threshold probabilities according to decision curve analysis, indicating that the use of our predictive nomogram for determining therapeutic strategies would obtain a better clinical outcome. Our nomogram provides a promising tool for assisting radiologists and physicians in the diagnosis of IFD of the lung, which will allow patients with IFD to undergo proper treatment as early as possible. A recent study [18] similar to our object proposed a clinical-radiomic nomogram with a great predictive ability to differentiate IFD from BP in patients with hematologic malignancies. The difference from ours is that their model was only applicable for patients with hematologic malignancies, whereas our model is applicable for a wider range of patients, and many clinical factors have been taken into their study, which is what we are going to do for further study in the future.

There were several limitations in our study. First, the number of patients in the test set was relatively small, and more patients from different centers will be needed to validate the accuracy of the model in the future. Second, the retrospective design of the study may have introduced potential biases in enrolling the participants. Third, the most recent patients enrolled in the training set were from several years ago; due to the lack of complete data from the First Affiliated Hospital of Army Medical University in recent years, newer patients were not available.

In conclusion, our proposed nomogram may provide a solution for the challenge that physicians face in identifying IFD at an early stage.

## Supplementary Information

**Additional file 1**. On the orininal data of the study.

## Data Availability

Some data generated or analysed during this study are included in the supplementary files. Full datasets are not publicly available due to the large amount of data but are available from corresponding authors on reasonable request.

## Abbreviations

AUC: Area under the ROC curve
BP: Bacterial pneumonia
BAL: Bronchoalveolar lavage
CI: Confidence interval
CT: Computed tomography
DCA: Decision curve analysis
EORTC/MSGERC: European Organization for Research and Treatment of Cancer and the Mycoses Study Group Education and Research Consortium
GGO: Ground glass opacity
GM: Galactomannan
GLCM: Gray-level cooccurrence matrix
GLDM: Gray-level dependence matrix
GLRLM: Gray-level run length matrix
GLSZM: Gray-level size zone matrix
ICCs: Intra-class and inter-class correlation coefficients
IFD: Invasive fungal disease
LASSO: Least absolute shrinkage and selection operator
MSE: Mean square error
MSG: Mycoses Study Group
NGLDM: Neighborhood gray-level dependence matrix
OR: Odds ratio
PACS: Picture archiving and communication system
Rad-score: Radiomics score
ROC: Receiver operating characteristic
SVM: Support vector machine
VOI: Volume of interest
RHS: Reverse halo sign
